# Gender equality and smoking among 15 to 25 year olds—a time-based ecological analysis of developments in Germany from 1960 to 2005

**DOI:** 10.3389/fpubh.2024.1295050

**Published:** 2024-02-16

**Authors:** Jana Roczen, Gabriele Bolte, Birgit Reineke, Ronny Kuhnert, Anne Starker, Emily Mena

**Affiliations:** ^1^Department of Social Epidemiology, Institute of Public Health and Nursing Research, University of Bremen, Bremen, Germany; ^2^Health Sciences Bremen, University of Bremen, Bremen, Germany; ^3^Department of Epidemiology and Health Monitoring, Robert Koch Institute, Berlin, Germany; ^4^Department of Prevention and Health Promotion, Institute of Public Health and Nursing Research, University of Bremen, Bremen, Germany

**Keywords:** smoking, smoking prevalence, gender equality, gender inequality, young adults, time-based, tobacco control, Germany

## Abstract

**Introduction:**

Smoking is a major risk factor for premature death and health problems in which there are significant gender differences in the prevalence of smoking. This ecological study examines the correlation between changes in gender equality and prevalence of smoking among young adults (15–25 years old) in Germany over a period of 45 years (1960–2005).

**Methods:**

Gender inequality was measured using the United Nations Gender Inequality Index (GII), which is composed of three dimensions; health, empowerment and labour market. It was calculated for the entire registered German population in five-year intervals with values between 0 and 1 (1 = highest inequality). The smoking prevalence of young women and men in Germany was established using a reconstruction method. A gender smoking ratio (GSR) with values between 0 and 1 was determined (1 = identical smoking prevalence among men and women). The smoking behaviour was illustrated and stratified by education. The correlation between the GII and the GSR was analysed.

**Results:**

The GII decreased from 0.98 to 0.56 between 1960 and 2005. The GSR increased from 0.34 to 0.93. There was a strong negative correlation between the GII and the GSR (*r* = −0.71). The strength of the correlation fell slightly as the level of education decreased. An increase in gender equality as measured by the GII came along with similarities of smoking prevalence between young women and young men.

**Conclusion:**

Successful tobacco prevention among young women and men may benefit from involving experts in gender-specific public health research to develop counter-advertising and gender-specific information as needed.

## Introduction

Smoking is one of the leading risk factors for premature deaths and health issues worldwide (1). Germany ranks ninth out of 195 countries in smoking prevalence among people aged 10 and older and 13th among the subpopulation of adolescents aged 15 to 19 (2). In 2019, 6.3% of the female and 8.0% of the male adolescents aged 12 to 17 years considered themselves smokers. In the age group 18 to 25, 23.7% of female and 33.4% of male young adults smoked (3, 4).

From a Public Health point of view, it is important that adolescents do not take up smoking in the first place (smoking initiation). In Germany, there are programmes and educational work by the Federal Centre for Health Education that are specifically designed to prevent young people from starting to smoke or to support them in quitting (5, 6). Young adults are likely to be more impulsive and self-confident than adults due to the neurobiological changes during this developmental phase (7). Thus, they are particularly vulnerable to harmful products that offer instant gratification, a sense of adventure or social status. Harmful products may pose a higher risk to young adults than to adults due to the rapid changes in the adolescent brain, for example through a higher likelihood of becoming addicted (7).

Tobacco industry advertising contradicts the idea of prevention. A report from Germany shows that young adults especially are susceptible to the perception of tobacco industry’s promotional activities (8). Despite bans on tobacco advertising, for example on television, radio or through print media or product placements, many tobacco advertising measures are still permitted in Germany. Outdoor and point-of-sale advertising, as well as advertising in cinemas after 6 pm and direct marketing for tobacco are currently allowed. As a consequence, young adults in Germany are inevitably exposed to tobacco advertising campaigns in a variety of settings (9).

The marketing strategies employed by the tobacco industry are also adapted to appeal to prevalent motivations for smoking among young adults. Tobacco advertising markets the use of tobacco products to achieve well-being, popularity, relaxation or companionship with tobacco products, for example. In addition, advertising specifically addresses gender issues among young adults (10). Thus, for boys the feeling of belonging to a peer group, to ‘be cool’ or to feel grown up seems to be a particularly prominent motivation for smoking. For girls, it is more often about weight reduction, attracting attention, rebelling against parents or teachers, and relaxation (11). Therefore, these differences in motivation and smoking behaviour are likely to be influenced by prevailing gender norms and roles. Gender is defined by a multidimensional social construct that is constantly changing and that characterises boys and girls, and men and women in their norms and roles within a group or society (12). Gender roles describe a construct where cultures and societies have expectations about the roles and behaviour of boys and girls and men and women which in turn promote gender-specific behaviour (13). Sex and gender differences can be seen in the socio-cultural use of tobacco products (“gender”) and in the biological reaction to tobacco consumption (“sex”). Both aspects interact with each other and influence smoking initiation as well as general smoking behaviour (e.g., currently smoking or not, frequency) and quitting behaviour (14).

The smoking behaviour of young women and men in Germany differs and has changed over the course of time depending on social status. In the past century, there has been a shift from higher smoking prevalence in higher to lower social status in Germany, which was observed earlier in young men than in young women (9). In this context, children and adolescents (11–17 years) hardly showed any differences in smoking behaviour between the sexes and the educational differences in smoking behaviour were similar for both sexes. Young adults (18–25 years) as well as adults (>25 years) on the other hand showed differences in smoking behaviour between genders with educational differences in smoking behaviour being similar for both genders (15, 16). Comparisons in other European countries also show that men with a lower educational status have a higher prevalence of smoking than those with a higher educational status. This gradient between the different education groups is more distinct in younger age groups, and is also a trend that is discernible among younger women (17–20).

Gender analyses in health, including smoking initiation and smoking behaviour, should examine the extent to which gender inequality influences health behaviours (21). Gender inequality exists when boys, girls, men, and women have unequal opportunities to achieve their potential, for example in terms of their health. This study investigates the temporal changes in gender inequality, measured by the United Nations Gender Inequality Index (GII), the prevalence of smoking, and a gender smoking ratio (GSR) among young adults (defined as the 15-25-year age group) in Germany between 1960 and 2005. A particular concern of the present study was to calculate smoking prevalence only among young adults who smoke tobacco cigarettes. In order to maintain theoretical and analytical accuracy, we consider it necessary to exclude products such as e-cigarettes, which have only been available on the European markets since 2006 (22). According to a survey conducted in 2006, 1.4% of respondents regularly used e-cigarettes at that time. Among smokers, 32.7% had ever tried e-cigarettes. Of those who had never smoked, 2.3% had ever tried e-cigarettes (23). Smoking products other than tobacco cigarettes are likely to be associated with different smoking behaviour in general, which in turn may influence gender-specific smoking patterns. At this stage, some gender differences in the prevalence of e-cigarettes compared to tobacco cigarettes can already be identified among young adults (22). The specific reference to tobacco cigarettes counteracts a possible bias that could result from the change in gender-specific smoking prevalence throughout the study period due to the introduction of tobacco-free smoking products at a later stage. The GII has mainly been used to compare different health contexts in different countries or populations, but it was also used for a regional gender differences in life expectancy in the European Union (24–28); however there is a paucity of studies on the temporal evolution of the index within a population, and the correlation between the GII and smoking behaviour in a country. This study determines the relationship between the GII and the GSR, considering also education as a stratifying factor to assess gender inequality in Germany and its association with the smoking behaviour of young women and men and to illustrate changes over time.

## Materials and methods

This ecological study is derived from two different data sources. The data on the prevalence of smoking, the GSR, the education level and the birth year based on the German Health Update study (GEDA) by the Robert Koch Institute, the national public health authority in Germany. The GEDA study is a representative survey of the German-speaking adult resident population in private households with a landline connection. The GEDA study, which is regularly repeated as part of health monitoring, is aimed at the continuous observation of developments in the incidence of disease and in health and risk behaviour and is intended to contribute to providing health reporting and health policy with timely information to identify health trends in the population or in population groups. For this current study, data from the surveys conducted in 2009, 2010 and 2012 of the GEDA were pooled, resulting in a total of 33,720 participants. The analyses are limited to 15 to 25-year-olds in each of the years studied. This resulted in a population between *n* = 9,425 and *n* = 14,000 in the years 1960–2005. The population of young men aged 15–25 years ranges between 3,968 and 6,755 in the years from 1960 to 2005. The population of young women at the same age is between 4,448 and 8,342 in the same time period.

The German Federal Statistical Office (Statistisches Bundesamt) provided the aggregated population data used for the calculation of the GII. This includes data on education and labour force participation based on the German micro-census. Data concerning maternal mortality is based on the cause-of-death statistics, while the fertility rate of adolescents was derived from the German population statistics. The data used to calculate the proportion of men and women in parliament was gathered from a data manual on the history of the German parliament (29).

### Gender smoking ratio

The prevalence of smoking for calculation of a GSR was determined using weighted data from the GEDA study, which examines the association between health and lifestyle of adults in Germany. The survey was conducted by means of telephone interviews (30–33). The study participants are representative for the German population aged 18 years and older. In this study the analyses are limited to 15 to 25-year-olds. All participants who completed the relevant items of the questionnaire were included in the analyses. Smoking status was categorised into non-smoker, current smoker and ex-smoker [Questionnaire scheme: Do you currently smoke—even if only occasionally? Current smokers (1 = Yes, daily and Yes, occasionally) were asked: How old were you when you started smoking, even if only occasionally? And what do you smoke? You could also give more than one answer. Ex-smokers (2 = No, no longer) were asked: Did you used to smoke once a day? And what did you smoke in the past? You can also give more than one answer. How old were you when you stopped smoking?]. Excluded from the analyses were participants who exclusively smoke cigars or pipes as they represent only a very low percentage of the German population (9). Participants who stated that they had been younger than 11 years old when they started or quit smoking were also excluded, as statistics in Germany on the prevalence of smoking often start at the age of 11. This means that the data can be directly compared. In Germany, it is also the case that children under the age of 11 attend elementary school and move on to secondary school at the age of 11 and are therefore exposed to different peer groups and different impressions.

The prevalence of smoking was reconstructed for each calendar year between 1960 and 2005 using the method introduced by Harris (34) to simulate the data. For this, each participant was assigned a smoking status (smoker/non-smoker) for each calendar year. Non-smokers are considered as such for the entire period, while current smokers are regarded as smokers from the year in which they started smoking until their current age. Former smokers are categorised as smokers from the time they started smoking until the time of quitting; before and after that time, they are counted as non-smokers. Smokers who did not answer when they took up smoking are assigned the average age of smoking initiation from their birth cohorts. Former smokers who did not indicate ever giving up smoking are classified as smokers until the end of this study period (2005).This means for example, that an individual smoker who reported in the 2010 survey that he or she was born in 1970 and smoked between the ages of 18 and 35 will have the following statuses: From 1981 to 1987 (ages 11–17), this person will be counted as a non-smoker. From 1988 to 2002, the person reported smoking and is therefore recorded as a smoker in these years. From 2002 until the end of the study period, the person is again classified as a non-smoker. In order to determine the prevalence of smoking for each calendar year between 1960 and 2005, the number of smokers was divided by the total population of 15-25-year olds in the corresponding calendar year (34).

To calculate a female-to-male GSR, the prevalence of smoking in young women was divided by the prevalence of smoking in young men. Values below 1 describe a higher prevalence of smoking in young men, value equal 1 means identical smoking prevalence among men and women, values above 1 correspond to a higher prevalence of smoking in young women (35).

### Educational status

The education data also comes from the GEDA data and are used for stratification in this study. Data on school and vocational education of the respondents was collected, in order to calculate the education groups according to the ISCED classification (International Standard Classification of Education) and categorised into low, middle and high educational status (36).

### Gender Inequality Index

The GII describes the extent to which the human development potential of a country is influenced by gender inequality (37). The index assumes values between 0 and 1, with values closer to 0 corresponding to less gender inequality and more human development potential (37). The index includes three dimensions: health, empowerment, and the labour market. The *health* dimension measures maternal mortality (per 100,000 live births) as well as adolescent birth rates (number of births per 1,000 women aged 15 to 19 years). The *empowerment* dimension consists of two indicators: the proportion of the population aged 25 and older with at least a secondary-level education and the distribution of female and male members of the parliament. The dimension *labour market* describes the labour force participation rates of males and females (ages 15 to 64 years) (37). A person is defined as employed when they are aged 15 years and over and (a) work at least 1 hour a week for remuneration, (b) are self-employed in a trade, or (c) work in a family business without being paid.

All indicators were generated in five-year intervals for the period from 1960 to 2005. Data on education was available for the years 1961, 1970, 1976, 1980, 1985, 1989, 1991, 1995, 2000, and 2005 and averaged over the adjacent values for the intervening periods. In order to calculate a GII for 1960, the data for education from the following year was used. For the period from 1960 to 1989, only indicators for the former Federal Republic of Germany (FRG) are provided by the German federal statistical office. Data from the former German Democratic Republic (GDR) were therefore not included in the analyses. As of 1990, the data include Germany as a whole. For the calculations of the GII, the requirements from the UN Human Development Report 2011 were applied (37).

### Statistical analyses

In this ecological study the temporal changes of GII and GSR as well as prevalence of smoking of 15-25-year-old young women and men over the period from 1960 to 2005 in Germany was illustrated using descriptive statistics. The smoking prevalence also was stratified by educational status and was descriptively presented from 1960 to 2005. Correlation between GSR and GII was assessed using Pearson correlation coefficient. A significance level of 0.05 was defined for the analyses. SAS^®^ 9.4 was used to conduct all analyses (SAS Institute Inc., Cary, NC, United States). All figures were created with Microsoft Office Excel 2007 (Microsoft Corporation, Redmond, WA, United States).

## Results

### Temporal trends in gender inequality index and its components

**Figure 1:**
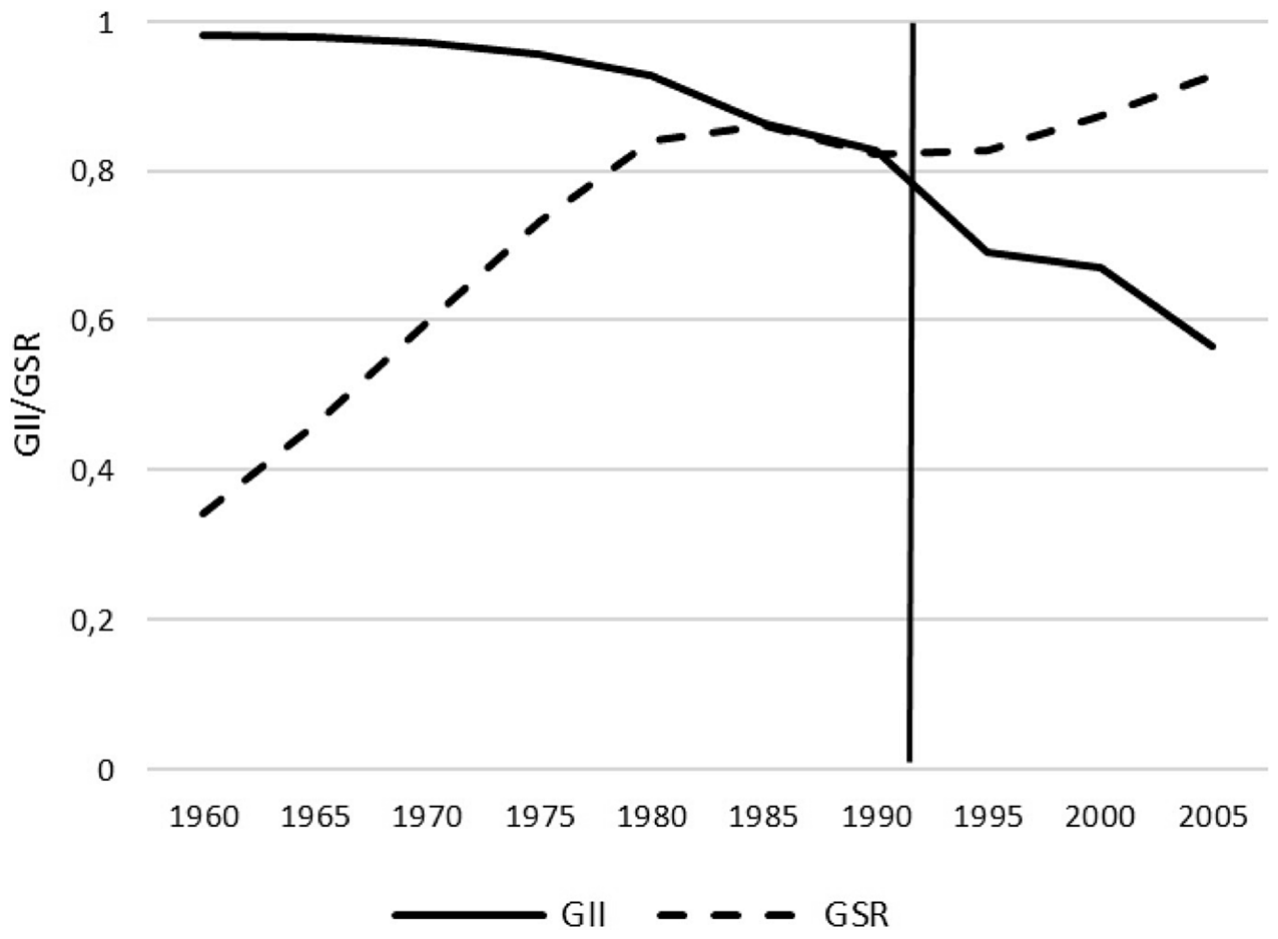
Gender inequality index (GII) and gender smoking ratio (GSR) from 1960 to 2005 in Germany.

The GII showed a decline from a maximum value of gender inequality at 0.98 to a minimum of 0.56 from 1960 to 2005 (Figure 1; Additional file 1).

**Figure 2:**
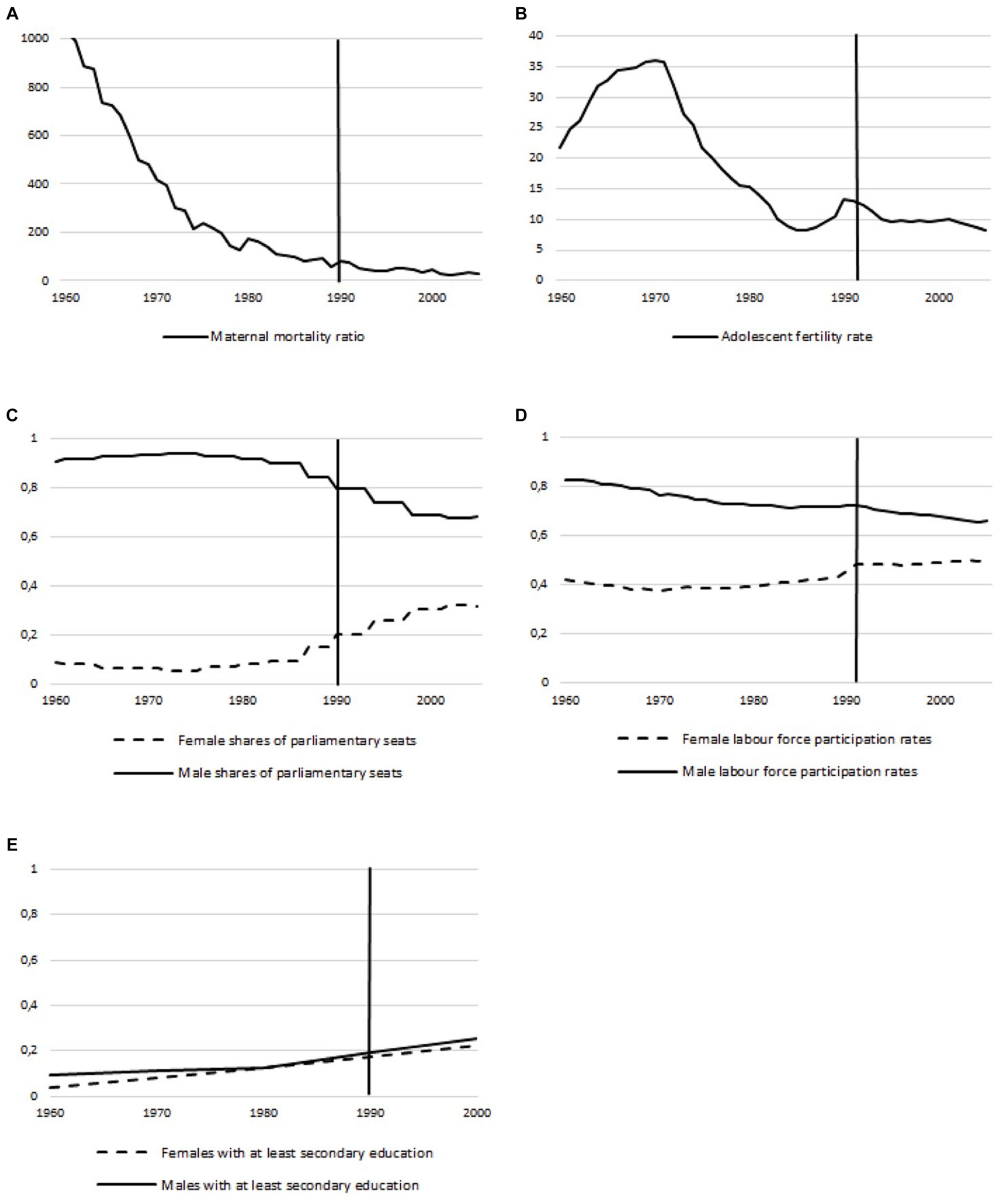
Temporal trends of the gender inequality index components from 1960 to 2005 in Germany: **(A)** Maternal mortality rate; **(B)** Adolescent fertility rate; **(C)** Share of parliamentary seats; **(D)** Labour force participation rates; **(E)** Population with at least secondary education. From 1960 to 1990: Former federal territory of Germany, since 1990: The Federal Republic of Germany (FRG).

The components of the GII showed the following trend: The **maternal mortality** per 100,000 live births decreased from 1,030 maternal deaths per 100,000 live births in 1960 to a maternal death rate of 28 per 100,000 in 2005. The **adolescent birth rate** initially increased from 1960 to 1970 from 22 to 36 births per 1,000 women aged between 15 and 19 years and then dropped to eight births per 1,000 women by 2005. The **proportion of female and male individuals with at least secondary education** was very similar and increased from less than 10% to almost 50% during the investigated time period. The proportion of women with at least secondary education was slightly lower than that of men throughout the entire study period. The **distribution of seats in the German parliament** showed a consistently large difference between men and women between 1960 and 1985, with the proportion of men between 90 and 94% and women, conversely, between 10 and 6%. From 1990, the proportion of men declined from around 80 to 68% in 2005. For women, a parallel increase to around 32% could be observed. The **female labour force participation** rate increased from 42 to 51% from 1960 to 2005. At the same time, the male labour force participation rate dropped from 82 to 66%. While labour participation among men in 1960 was about twice as high as the rate of women, in 2005 about one third more men than women worked (Figure 2; Additional file 2).

### Smoking prevalence and gender smoking ratio

**Figure 3:**
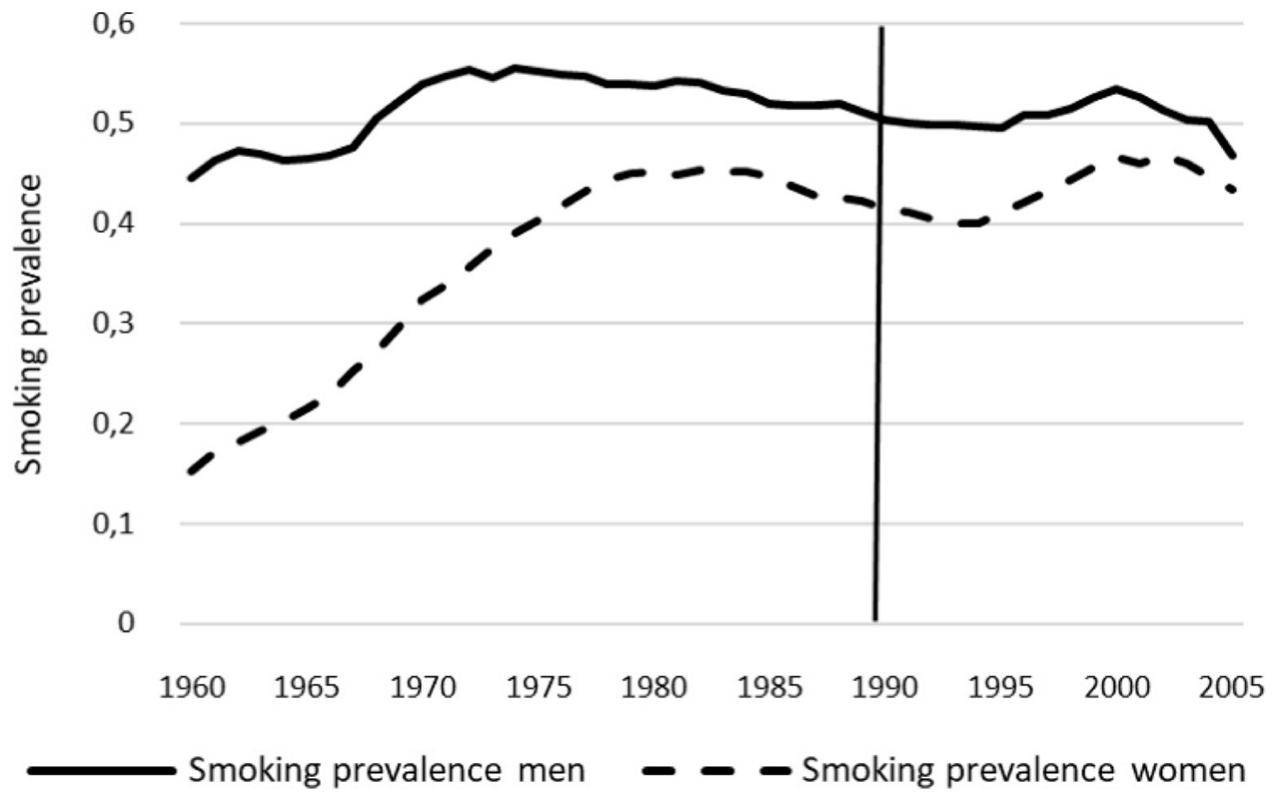
Smoking prevalence of young women and men from 1960 to 2005 in Germany.

The GSR increased continuously from 0.34 in 1960 to 0.93 in 2005 (Figure 1; Additional file 1). Over this monitored period, the prevalence of smoking among young women approached that among young men. Overall, there are fluctuations of about 10% in the smoking prevalence of young men between 1960 and 2005. In young men, the prevalence of smoking increased from 45% in 1960 to 55% in 1975. After that, it briefly remains constant and then declines to 50% after 10 years. Until 2004, the value fluctuates slightly between 49 and 52% and then drops to 47% in 2005. The prevalence of smoking among young women tripled from 15 to 45% from 1960 to 1985. The smoking prevalence then decreases to 40% until 1994 and before rising again in the following 10 years to 46%. In the last 2 years of the studied period, the prevalence of smoking is approximately 44% (Figure 3; Additional file 1).

### Smoking prevalence by education

**Figure 4:**
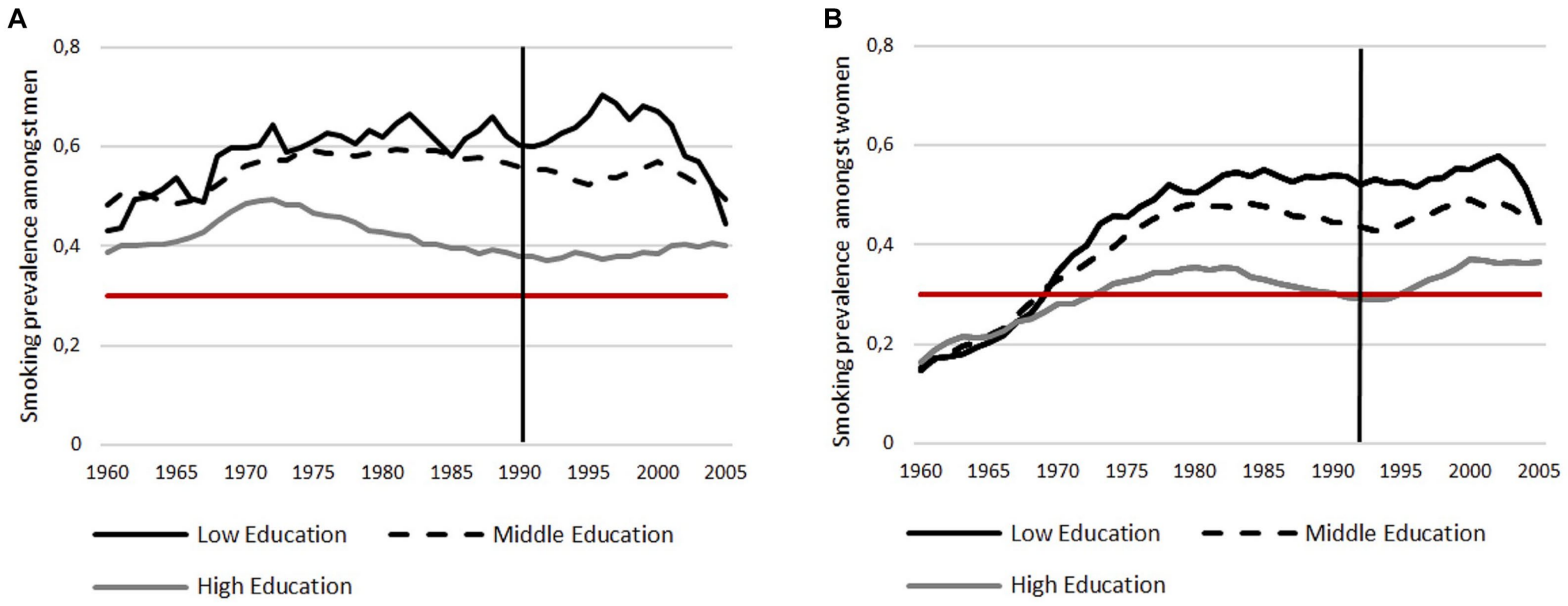
Smoking prevalence of men and women stratified by education: **(A)** Smoking prevalence among men; **(B)** Smoking prevalence among women. For better illustration, a reference line with 30% is added.

Among both, young women and men, smoking prevalence increases with decreasing educational level. In all education groups, the prevalence of smoking is lower among young women than among young men. The smoking behaviour of young men was constant during the period being examined: Young men with a low and middle educational status smoked consistently more than young men with a higher educational status. From 1969 onwards, a consistent picture is evident: the higher the educational status, the lower the smoking prevalence. From the year 2000 onwards, the smoking prevalence of young men with a low educational status decreases and falls below that of young men with a middle educational status in the last years of the study period, whereas prevalence in young men with a high educational status remained the lowest (Figure 4). The smoking prevalence of young women stratified by education showed that young women with a high level of education had the highest smoking prevalence at the beginning of the study period, before the pattern reversed after 10 years of the study period and the highest smoking prevalence was among young women with a low level of education, followed by middle education and, lastly, highly educated. From 2003 onwards, the smoking prevalence of young women with a low educational status decreases and approaches the smoking prevalence of young women with a middle educational status, whereas prevalence in young women with a high educational status remained largely unchanged (Figure 4; Additional file 3).

### Correlation between Gender Inequality Index and gender smoking ratio

The bivariate correlation between the GSR and the GII showed a strong correlation of −0.71 (95% CI: −0.93, −0.15; Additional file 4); indicating that more gender equality correlates with greater equality in the smoking behaviour between young women and men. The greater equality in smoking was due to the fact, that young women’s smoking rate approached the rate of male smokers. The strength of the correlation decreases slightly as the level of education decreases [low educational status: *r* = −0.69 (95% CI: −0.92, −0.10); middle educational status: *r* = −0.74 (95% CI: 0.93, −0.20); high educational status: *r* = −0.78 (95% CI: −0.94, −0.29); Additional file 4].

## Discussion

The results of our ecological study illustrate how gender equality in Germany has increased in the period from 1960 to 2005 and in parallel the GSR has decreased. This trend is based on the fact that during the period under study, the prevalence of smoking increased among women while it remained relatively constant among men, which in turn led to a decrease in GSR (Figures 1, 3). Another core finding was that young women and men with a higher educational status smoked less than those with a middle and low educational status. The strong negative correlation between the GSR and the GII shows that more gender equality correlates with greater equality in the smoking behaviour between young women and men in Germany.

According to a 2019 study by the European Institute for Gender Equality, Germany is still below the European Union average in terms of gender equality. Although there has been a slight increase in gender equality since 2015, Germany ranked 12th in comparison to the other European member states in 2019 (38). This is in spite of the fact that the Federal Constitution has prescribed equal rights for men and women in Germany since 1949. The article which defines these rights includes promoting the enforcement of equal rights for women and men as well as efforts to eliminate existing disadvantages by the government (39). Nevertheless, unequal chances for men and women are reflected in unequal social, economic, and political participation and promote discrimination, violent conditions and structural disadvantages due to institutional frameworks. In 2018, new equality policy goals for Germany were published by the Federal Ministry (40).

Our findings of social differences in smoking are in line with similar findings in other European countries (17–20, 35, 41). In the beginning of the 20th century, women rarely smoked because it was socially undesirable or unacceptable. This is reflected in the social value systems of the time and gender-specific defined roles (42–44). The change in smoking behaviour mirrors the social change in gender roles and identities during the 20th century. The emancipation movement over the past 100 years, for example, was accompanied by an increased acceptance of women smoking. The tobacco industry took advantage of this early on and introduced gender-specific tobacco advertising, using the image of a woman smoking as a sign of emancipation for its marketing campaigns (14, 39). Later, the tobacco industry’s advertising campaigns and marketing strategies aimed at young women in privileged circumstances, were shaped not only by notions of independence but also of romance and glamour, leading to a higher prevalence of smoking in this age group (41–44). However, meanwhile, it is particularly noteworthy that in several European countries including Germany, the highest smoking rates are now seen in girls with lower social status (41, 45).

The results of our study confirm that gender equality correlates with greater equality in smoking behaviour between young women and men. The promotion of gender equality in a society should therefore be taken into account from a political perspective when developing anti-smoking messages and counteract the targeted gender-based advertising strategies of the tobacco industry.

The total annual tobacco advertising expenditure has increased in recent years from approximately 193 million Euros in 2008 to 210 million Euros in 2019 (3, 46). Advertising for tobacco products, especially aimed at the target group of young adults, is extremely lucrative, as several studies also show that a quarter to half of young adults who start smoking stick with it and become daily smokers (11, 21, 25). In Germany, the tobacco industry currently still has many possibilities to promote its products. The restrictions on tobacco advertising have been extended since the beginning of 2021. Since then, cinema advertising for tobacco products is only allowed at certain times and for films suitable for 18-year olds and over. From 2022, advertising is only allowed in specialised shops (47). Notwithstanding the fact that the overall gender inequality measured by the GII is significantly higher in Germany than in Spain, a similar trend of an increase in gender equality and a simultaneous decrease in GSR was observed by Bilal and colleagues who examined the relationship between the GII and GSR for the entire Spanish population (35). In contrast to Bilal and colleagues, whose analyses did not focus on a specific age group, we have limited our analysis to the subgroup of 15 to 25-year olds, as this population might have a high potential for smoking prevention. It might be noteworthy, that although the meaning of gender roles may become manifest further in life, the correlation between the GII and the GSR in Germany could also be observed in this age group of young adults.

The GII was developed by the United Nations to compare countries around the world (37). However, it should be noted that the GII includes components, such as maternal mortality, that may not fully capture gender inequality in the industrialised nations, like Germany. In other nations with poorer healthcare, these indicators are more meaningful. As a single indicator, therefore, maternal mortality cannot be considered a valid substitute for the GII. Nevertheless, we have chosen this index to ensure the best possible comparability with other studies worldwide. Particularly with regard to the other individual indicators of the GII, such as the labour force participation rate, the single indicators of the GII can certainly be regarded as valid proxies of the GII. As shown in Figure 2, both access to (higher) education and the number of parliamentary seats held by women in Germany have increased steadily over time. In principle, it is encouraging from a gender equality perspective that the opportunities for greater female labour force participation are steadily improving, but higher labour force participation can also be associated with more work stress in everyday working life, which in turn leads to a higher prevalence of smoking (48), which is also reflected in the gender smoking ratio.

### Limitations and strength

Some limitations need to be addressed. As already mentioned, not all GII indicators reflect the GII to the same extent for Germany. Furthermore, reproductive health, is a very important factor in mapping women’s health. In contrast, however, no information on men’s health status is included in the calculation of the GII. To obtain a comprehensive picture of gender inequality, this would potentially be a relevant factor (42). Furthermore, for the present analyses, it must be considered when interpreting the GII for Germany that from 1960 to 1989 only data for the former federal territory are available, and from 1990 this data is for Germany as a whole. It might have been worthwhile to calculate the GII for the entire period for Germany as a whole or to conduct comparative analyses between GDR and FRG. Due to the different structures and political systems of GDR and FRG, a comparison of these societies especially in relation to aspects of gender inequality might be very insightful. Many discriminatory laws in the GDR were repealed in 1949, much earlier than in the FRG. An example concerning gender inequalities are the different employment rates of women between the GDR and FRG. Female workers were urgently needed in the GDR which resulted in a female employment rate of 45% in 1950 and an increase to over 90% in 1989 (49). However, the household chores were assigned in most cases to women, which led to a double burden and often prohibited career advancement. Additionally, the proportion of female university students was lower than in the FRG (49). Finally, another example is the proportion of female policy-makers in the government which was significantly higher in the GDR than in the FRG. While a quarter of policy-makers were women in the GDR in 1960, the proportion in the FRG was only 9%. In 1989, the proportion of women in the GDR government was 32%, while in the FRG it was approximately 15% (50, 51). However, the proportion of women in the higher, more powerful positions in politics was very low in the GDR (49). Consequently, it could be assumed that if data from the GDR were included, the GII would possibly be lower and there would therefore be less measured gender inequality.

Another limitation concerns the data used for the GSR. The sample of the GEDA study comprises the adult German-speaking population from private households in Germany based on a pool of publicly available telephone numbers from landlines, which means that people without a landline connection are excluded (30–33). This may introduce bias, as people without a landline connection are not captured. However, over 90 percent of households in Germany had a landline connection during the survey period (52). Furthermore, the calculation of smoking prevalence is based on self-reported smoking data. This may be subject to recall or social desirability bias.

In addition, the ecological study design does not allow conclusions to be drawn at the individual level but is limited to analyses at the population level.

To assess temporal trends a period of 45 years was analysed. Strength of our study is the large sample size and high quality of the data, which made it possible to provide valid and representative information about the 15-25-year-old residing in the former federal territory of Germany from 1960 to 1990 and for Germany as a whole from 1990 to 2005. The methodology used in this paper (by Harris et al.) allows for analyses over a long period of time, which is a strength compared to conventional ecological studies. To the best of our knowledge, it is the first study to show the temporal changes in gender inequality and smoking prevalence of young women and men between 1960 and 2005 in Germany.

## Conclusion

This study provides relevant information on the temporal development of smoking prevalence among young adults in Germany. It is the first ecological study to describe differences in smoking behaviour in Germany as a function of educational status over a period of several decades. In terms of monitoring the development of gender equality in a society, gender-specific smoking patterns might be predicted more accurately and tobacco control measures could be adapted accordingly. Experts in gender-sensitive public health research should be involved and consulted in the development of counter-advertising messages and gender-specific information in light of tobacco prevention in young women and men.

## Data availability statement

The original contributions presented in the study are included in the article/Supplementary material, further inquiries can be directed to the corresponding author.

## Author contributions

JR: Conceptualization, Methodology, Writing – review & editing, Formal analysis, Project administration, Writing – original draft. GB: Conceptualization, Methodology, Writing – review & editing. BR: Writing – review & editing, Data curation, Formal analysis. RK: Writing – review & editing, Conceptualization, Methodology, Resources. AS: Conceptualization, Methodology, Resources, Writing – review & editing. EM: Conceptualization, Methodology, Writing – review & editing, Supervision.
