# Supplementary material for: Gender equality and smoking among 15 to 25 year olds—a time-based ecological analysis of developments in Germany from 1960 to 2005
Source: Front Public Health. 2024 Feb 16;12:1295050. doi: 10.3389/fpubh.2024.1295050 (PMC10904588; doi:10.3389/fpubh.2024.1295050)
Supplement: Supplementary file 4 [file Table_4.DOCX]

**Additional file 4. Correlation between Gender Inequality Index (GII) and Gender smoking ratio (GSR), stratified by education.**

|  | Pearson correlation coefficient (95% CI) |
| --- | --- |
| Total | -0.71 (-0.93, -0.15) |
| Low educational status | -0.69 (-0.92, -0.10) |
| Middle educational status | -0.74 (-0.93, -0.20) |
| High educational status | -0.78 (-0.94, -0.29) |
